# The Impact of Rurality on Substance Use and Adverse Mental Health Among High School Students in Vermont, USA: A Cross-Sectional Study

**DOI:** 10.7759/cureus.103767

**Published:** 2026-02-17

**Authors:** Olivia Tarmey, Gina Neidig, Anna Cosentino, Natalie Hewitt, Chike Asanya, Thomas V Delaney, Elzerie De Jager

**Affiliations:** 1 Larner College of Medicine, University of Vermont, Burlington, USA; 2 Department of Pediatrics, University of Vermont, Burlington, USA; 3 Department of Medicine, University of Vermont, Burlington, USA

**Keywords:** adolescent, mental health, rural, substance use, suicide

## Abstract

Introduction

Rates of poor mental health among high school students in the United States are increasing. Both substance use and rurality are independently associated with poor mental health outcomes among adolescents. This study explores the impact that rurality may have on the relationship between substance use and poor mental health outcomes among Vermont high schoolers.

Methods

The 2021 Vermont Youth Risk Behavior Survey (YRBS) was used (N = 15,097). Multivariate logistic regression was used to assess the relationship between adverse mental health and substance use by rurality.

Results

Experiences of adverse mental health were associated with substance use behaviors among rural and non-rural populations. The associations between adverse mental health and substance use variables did not significantly vary by rurality.

Conclusions

Rural environments may not modify the relationship between adverse mental health and substance use. Future research is needed to understand the causal mechanisms between adverse mental health and substance use and how these may differ by rurality.

## Introduction

Mental health among high school students in the United States is worsening, with increasing rates of suicidal ideation, suicide attempt, and persistent sadness and hopelessness [1]. Substance use (including tobacco, cannabis, and alcohol) is associated with poor mental health, including depression, anxiety, and suicidality [2-4]. There is no consensus on how rurality may influence the relationship between mental health and substance use [5-8]. Data support that both adverse adolescent mental health and substance use may be influenced by living in a rural or urban setting. Specifically, individuals living in rural areas experience higher rates of substance use, suicidal ideation, and suicide [5,8-12]. Despite associations between substance use and adverse mental health, rurality and adverse mental health, and rurality and increased substance use, current literature has yet to examine the modifying effect that rurality may have on the relationship between substance use and adverse mental health among adolescents. Illuminating the relationship between substance use and adverse mental health outcomes in the context of rural versus urban settings is important to inform future substance use and mental health interventions and programming for adolescents.

This research project examines whether living in a rural area modifies the relationship between substance use and adverse mental health outcomes (suicidal ideation, suicide attempt, depression and anxiety symptoms, and self-harm) among high school students in Vermont.

## Materials and methods

We conducted a cross-sectional study using data from the Vermont Youth Risk Behavior Survey (YRBS) questionnaire. The state of Vermont was selected due to its high proportion of adolescents residing in rural areas. Data were collected between September and December of 2021 [13]. Observations with missing data were excluded.

Rurality was coded dichotomously using county-level Rural-Urban Commuting Area Codes (RUCA), with RUCA 4-10 (micropolitan, small town, and rural) coded as rural and 1-3 (metropolitan central and metropolitan outlying) coded as non-rural. A county was considered non-rural if all towns in that county were coded as 1-3 [14]. Substance use was defined as current substance use and lifetime illicit use. Current substance use included respondents who reported using alcohol, cigarettes, e-cigarettes, or marijuana in the last 30 days. Lifetime illicit use included respondents who reported ever using cocaine, heroin, methamphetamine, or prescription pain medication without a doctor’s prescription or differently than prescribed. Adverse mental health variables included self-harm and suicide attempt within the last 12 months and past 30-day anxiety and mental health status. Substance use variables were collapsed into binary categories based on the timeframe of reference in each question (ever versus last 30 days) to avoid data suppression.

Covariates included the following: age, sex, race, gender identity, and sexual orientation. Age was aggregated into three categories: <12-14, 15-16, and 17+. Race was grouped into two categories: Black, Indigenous, and People of Color (BIPOC) and White (students who identified as White and not Hispanic or Latino). BIPOC included students who identified as Hispanic or Latino, American Indian or Alaska Native, Asian, Black or African American, Native Hawaiian, or Other Pacific Islander. Gender identity and sexual orientation were grouped into two categories: LGBTQ+ (students who identified as gay or lesbian, bisexual, or questioning or described their sexual identity in another way and students who identify as transgender or are not sure if they are transgender) and heterosexual and not transgender.

Chi-square tests were used for descriptive statistics. Multivariate logistic regression was used to examine associations between the adverse mental health variables and substance use variables among rural and non-rural populations. Models were adjusted for age, sex, race, and sexual orientation and gender identity. An alpha level of 0.05 was used, and analyses were conducted using SPSS version 29 (IBM Corp., Armonk, NY). The weighting variable (overall analysis weight) was used for all analyses. This project was exempted from Institutional Review Board review.

## Results

The analysis included 15,097 observations. The respondents were a majority White, non-Hispanic (n = 12,693, 84.1%), and heterosexual and not transgender (n = 10,872, 72.0%) and lived in a rural county (n = 11,026, 73.0%). Nearly one in 10 (n = 1,293, 8.6%) reported lifetime illicit use, and one-third (n = 5,037, 33.4%) reported current substance use. The non-rural sample had a significantly higher proportion of BIPOC students (n = 830, 20.4%, versus n = 1,574, 14.3%; P < 0.001). The rural sample had a higher proportion of students reporting lifetime illicit use (n = 981, 8.9%, versus n = 312, 7.7%; P < 0.05), current use (n = 3,828, 34.7%, versus n = 1,209, 29.7%; P < 0.001), past-year suicide attempt (n = 793, 7.2%, versus n = 211, 5.2%; P < 0.001), past-year self-harm (n = 2,524, 22.9%, versus n = 841, 20.7%; P < 0.005), and past 30-day anxiety most of the time or always (n = 4,120, 37.4%, versus n = 1,350, 33.2%; P < 0.001) (Table 1). Interaction terms were tested between rurality and each mental health variable for each substance use outcome and were not significant.

**Table 1:** Sample Demographics -: Cell intentionally left blank BIPOC: Black, Indigenous, and People of Color

Variables	Total number (%)	Rural number (%)	Non-rural number (%)	p
Total	15,097	11,026 (73.0)	4,071 (27.0)	-
Age (years)	-	-	-	-
<12-14	3,447 (22.8)	2,528 (22.9)	919 (22.6)	0.558
15-16	7,806 (51.7)	5,672 (51.4)	2,134 (52.4)	-
>17	3,844 (25.5)	2,826 (25.6)	1,018 (25.0)	-
Sex	-	-	-	-
Male	7,528 (49.9)	5,455 (49.5)	2,073 (50.9)	0.115
Female	7,569 (50.1)	5,571 (50.5)	1,998 (49.1)	-
Race	-	-	-	-
BIPOC	2,404 (15.9)	1,574 (14.3)	830 (20.4)	<0.001
White and non-Hispanic	12,693 (84.1)	9,452 (85.7)	3,241 (79.6)	-
Sexual orientation and gender identity	-	-	-	-
LGBTQ+	4,225 (28.0)	3,062 (27.8)	1,163 (28.6)	0.337
Heterosexual and not transgender	10,872 (72.0)	7,964 (72.2)	2,908 (71.4)	-
Lifetime illicit substance use	-	-	-	-
Yes	1,293 (8.6)	981 (8.9)	312 (7.7)	0.017
No	13,804 (91.4)	10,045 (91.1)	3,759 (92.3)	-
Past 30-day alcohol, cigarette, e-cigarette, and/or marijuana use	-	-	-	-
Yes	5,037 (33.4)	3,828 (34.7)	1,209 (29.7)	<0.001
No	10,060 (66.6)	7,198 (65.3)	2,862 (70.3)	-
Attempted suicide in the last 12 months	-	-	-	-
Yes	1,004 (6.7)	793 (7.2)	211 (5.2)	<0.001
No	14,093 (93.3)	10,233 (92.8)	3,860 (94.8)	-
Self-harmed in the last 12 months	-	-	-	-
Yes	3,365 (22.3)	2,524 (22.9)	841 (20.7)	0.003
No	11,732 (77.7)	8,502 (77.1)	3,230 (79.3)	-
Current mental health	-	-	-	-
Never/rarely poor	5,279 (35.0)	3,790 (34.4)	1,489 (36.6)	0.018
Sometimes poor	4,537 (30.0)	3,315 (30.1)	1,222 (30.0)	-
Always/most of the time poor	5,281 (35.0)	3,921 (35.6)	1,360 (33.4)	-
Anxiety	-	-	-	-
Never/rarely anxious	4,980 (33.0)	3,604 (32.7)	1,376 (33.8)	<0.001
Sometimes anxious	4,647 (30.8)	3,302 (29.9)	1,345 (33.0)	-
Anxious always/most of the time	5,470 (36.2)	4,120 (37.4)	1,350 (33.2)	-

Lifetime illicit use

All mental health variables were significantly associated with lifetime illicit use among both rural and non-rural samples. Among rural participants, having self-harmed in the last year (OR = 4.36 {95% CI: 3.75, 5.06}), having attempted suicide in the last year (OR = 6.00 {95% CI: 5.05, 7.13}), experiencing poor mental health sometimes (OR = 1.40 {95% CI: 1.13, 1.72}) and always or most of the time in the last 30 days (OR = 3.41 {95% CI: 2.83, 4.13}), and experiencing anxiety sometimes (OR = 1.42 {95% CI: 1.14, 1.76}) and always or most of the time in the last 30 days (OR = 3.94 {95% CI: 2.99, 4.43}) were significantly associated with reporting lifetime illicit substance use (Table 2).

**Table 2:** Multivariate Logistic Regression

		Lifetime illicit substance use				Current alcohol, cigarette, e-cigarette, and/or marijuana use			
		Rural		Non-rural		Rural		Non-rural	
		OR (95% CI)	p	OR (95% CI)	p	OR (95% CI)	p	OR (95% CI)	p
Self-harmed in the last 12 months	No	Reference	-	Reference	-	Reference	-	Reference	-
	Yes	4.36 (3.75-5.06)	<0.001	3.99 (3.07-5.19)	<0.001	4.09 (3.70-4.54)	<0.001	3.66 (3.06-4.39)	<0.001
Attempted suicide in the last 12 months	No	Reference	-	Reference	-	Reference	-	Reference	-
	Yes	6.00 (5.05-7.13)	<0.001	6.85 (4.95-9.47)	<0.001	5.64 (4.77-6.67)	<0.001	5.29 (3.89-7.20)	<0.001
Current mental health	Never/rarely poor	Reference	-	Reference	-	Reference	-	Reference	-
	Sometimes poor	1.40 (1.13-1.72)	0.002	1.47 (1.02-2.11)	0.038	2.00 (1.79-2.23)	<0.001	1.64 (1.36-1.98)	<0.001
	Always/most of the time poor	3.41 (2.83-4.13)	<0.001	3.73 (2.41-4.71)	<0.001	3.67 (3.28-4.11)	<0.001	2.84 (2.35-3.44)	<0.001
Anxiety	Never/rarely anxious	Reference	-	Reference	-	Reference	-	Reference	-
	Sometimes anxious	1.42 (1.14-1.76)	0.001	1.51 (1.05-2.17)	0.027	1.83 (1.63-2.05)	<0.001	1.76 (1.46-2.12)	<0.001
	Anxious always/most of the time	3.94 (2.99-4.43)	<0.001	3.29 (2.32-4.65)	<0.001	3.58 (3.19-4.01)	<0.001	3.09 (2.53-3.76)	<0.001

Among the non-rural participants, variables associated with lifetime illicit use were having self-harmed in the last year (OR = 3.99 {95% CI: 3.07, 5.19}), having attempted suicide in the last year (OR = 6.85 {95% CI: 4.95, 9.47}), experiencing poor mental health sometimes (OR = 1.47 {95% CI: 1.02, 2.11}) and always or most of the time in the last 30 days (OR = 3.73 {95% CI: 2.41, 4.71}), and experiencing anxiety sometimes (OR = 1.51 {95% CI: 1.05, 2.17}) and always or most of the time in the last 30 days (OR = 3.29 {95% CI: 2.32, 4.65}) (Table 2).

Current substance use

All adverse mental health variables were significantly associated with current substance use among both rural and non-rural samples. Among rural participants, having self-harmed in the last year (OR = 4.09 {95% CI: 3.70, 4.54}), having attempted suicide in the last year (OR = 5.64 {95% CI: 4.77, 6.67}), experiencing poor mental health sometimes (OR = 2.00 {95% CI: 1.79, 2.23}) and always or most of the time (OR = 3.67 {95% CI: 3.28, 4.11}) in the last 30 days, and experiencing anxiety sometimes (OR = 1.83 {95% CI: 1.63, 2.05}) and always or most of the time (OR = 3.58 {95% CI: 3.19, 4.01}) in the last 30 days were significantly associated with current alcohol, cigarette, e-cigarette and/or marijuana use (Table 2).

Among the non-rural participants, variables associated with current alcohol, cigarette, e-cigarette, and/or marijuana use were having self-harmed in the last year (OR = 3.66 {95% CI: 3.06, 4.39}), having attempted suicide in the last year (OR = 5.29 {95% CI: 3.89, 7.20}), experiencing poor mental health sometimes (OR = 1.64 {95% CI: 1.36, 1.98}) and always or most of the time in the last 30 days (OR = 2.84 {95% CI: 2.35, 3.44}), and experiencing anxiety sometimes (OR = 1.76 {95% CI: 1.46, 2.12}) and always or most of the time in the last 30 days (OR = 3.09 {95% CI: 2.53, 3.76}) (Table 2).

## Discussion

Rurality did not modify the relationship between mental health and substance use among Vermont youth. Students living in rural areas with poor mental health had a similar risk of using substances, compared to students living in urban areas and vice versa.

Similar to prior research, we observed a consistent, positive relationship between mental health variables and substance use variables in both urban and rural cohorts [2-5]. Past-year suicide attempt had the strongest association with substance use in both rural and non-rural populations, even when controlling for demographic variables. This finding is in alignment with current literature that highlights the link between adolescent suicidality and an increased risk for substance use [4].

These results support the importance of robust mental health screening programs in high schools and connecting students with appropriate mental health and substance use prevention resources [15]. Moreover, these findings provide insights regarding the relationship between specific substances, substance use behaviors, and mental health risk factors among high schoolers in a predominantly rural state in the Northeastern United States. Further research is needed to examine these associations and the causal mechanisms behind these relationships.

Several limitations were noted. Specific substance use was collapsed into aggregate substance use variables. Examining substances individually may be of benefit. The rural and non-rural regions were defined at the county level, as this was the geographic level available in the dataset. Some counties may include mixed rural/urban contexts. Defining rurality using more granular geographic units (e.g., census tracts or blocks) may be beneficial. Covariates were collapsed to increase the power of our models; doing so may mask covariate subgroup differences. Socioeconomic status and access to resources may influence mental health outcomes but were not available in the dataset for covariate analysis. Despite being an anonymous survey, the YRBS participants may have underreported mental health, substance use, or other challenges, impacting the reliability and validity of the study findings [14-16]. Data were collected during the COVID-19 pandemic, where there was an increase in anxiety and depression across populations [17]. COVID-19 restrictions (e.g., temporarily closing treatment centers) and innovations (e.g., increased access to telehealthcare) could have improved or worsened the available resources for both rural and non-rural students.

## Conclusions

Despite known associations between adverse mental health outcomes and substance use among youth, research is inconclusive about the impact that living in a rural area may have on this relationship. Our results highlight how experiences of adverse mental health are associated with substance use behaviors among rural and non-rural populations in Vermont. Associations between mental health and substance use variables did not vary by rurality. Future research is needed to examine the causal mechanisms that underlie the relationship between rurality, substance use, and adverse mental health outcomes, to inform policies that support mental health and improve substance use prevention and treatment programs for youth.
